# Post-Transplant HCC Recurrence and Survival: Impact of Bridging Therapy and Tumor Biology in 185 Consecutive Liver Transplants

**DOI:** 10.3390/jcm15124464

**Published:** 2026-06-09

**Authors:** Bengt Arne Wiemann, Clara Antonia Weigle, Matea Basic, Julian Palzer, Philipp Tessmer, Oliver Beetz, Dennis Kleine-Döpke, Ulf Kulik, Nicolas Richter, Florian Wolfgang Rudolf Vondran, Moritz Schmelzle, Felix Oldhafer

**Affiliations:** 1 Department of General, Visceral and Transplant Surgery, Hannover Medical School, 30625 Hannover, Germany; bwiemann@ukaachen.de (B.A.W.); cweigle@ukaachen.de (C.A.W.); richter.nicolas@mh-hannover.de (N.R.);; 2 Department of General, Visceral, Pediatric and Transplant Surgery, University Hospital RWTH Aachen, Pauwelsstraße 30, 52074 Aachen, Germany

**Keywords:** liver transplantation, hepatocellular carcinoma, recurrence of hepatocellular carcinoma, transplant oncology

## Abstract

**Background:** Hepatocellular carcinoma (HCC) is a leading indication for liver transplantation (LT), representing a curative treatment option for selected patients. A remaining clinical challenge is the recurrence of HCC after transplantation, impacting long-term graft and patient survival. The impact of different bridging therapies (BTs) such as transarterial chemoembolization (TACE), local ablation or liver resection on recurrence rates remains unclear. We assessed post-transplant HCC recurrence and survival focusing on the role of pre-transplant bridging therapies. **Methods:** Adult recipients undergoing LT for HCC at Hannover Medical School from January 2007 to September 2022 were retrospectively analyzed (*n* = 185). Recurrence was defined as confirmed intra or extrahepatic HCC after LT. Overall survival (OS) and recurrence-free survival (RFS) were analyzed using Kaplan–Meier estimation and log-rank testing; multivariable Cox proportional hazards regression was used to identify independent factors influencing OS. **Results:** Pre-transplant BT was administered in 85.4% of patients, consisting of only TACE, (*n* = 20; 10.8%), local ablation, (*n* = 32; 17.3%), liver resection (*n* = 27; 14.6%) or a multimodal approach (*n* = 50; 27%). Post-transplant HCC recurrence rate was 9.2% with a median time to recurrence of 845 days (range 126–3978 days). Patients with post-transplant HCC recurrence had a significantly higher prevalence of viral hepatitis (70.6% vs. 57.1%; *p* = 0.01), higher pre-transplant AFP peak levels (37.5 vs. 10 ng/mL; *p* = 0.03), larger tumor sizes (median 3.95 cm vs. 2.6 cm; *p* = 0.03) and more poorly differentiated tumors (G3; 25.0% vs. 5.3%, *p* = 0.04). Kaplan–Meier analysis showed significant overall differences in OS and RFS among bridging therapy groups (*p* = 0.03). In the subgroup of early HCC < 3 cm, local ablation was associated with significantly improved OS compared to TACE (*p* = 0.035). Last measured pre-transplant AFP < 15 ng/mL was a significant predictor of both improved OS (*p* = 0.006) and RFS (*p* = 0.008), whereas peak AFP did not reach significance after correction. Multivariable Cox regression revealed HCC recurrence, high recipient BMI and low LabMELD as independently associated with reduced OS after LT. Median OS after HCC recurrence was 13 months. **Conclusions:** Our monocentric retrospective data indicate that post-transplant HCC recurrence is uncommon but remains a challenge regarding life expectancy and is influenced by pre-transplant bridging therapy. In the subgroup of early HCC < 3 cm, local ablation was associated with significantly improved OS compared to TACE. Last measured pre-transplant AFP < 15 ng/mL was associated with both improved OS and RFS, suggesting that treatment response may also represent a prognostically relevant factor. Further prospective validation of contemporary locoregional and systemic bridging approaches, especially in the context of tumor biology and treatment response, is warranted.

## 1. Introduction

Over the past decades, liver transplantation (LT) has evolved from an experimental procedure to the established standard of care for end-stage liver disease of both acute and chronic etiology. While extrahepatic malignancy is generally a contraindication to transplantation, hepatocellular carcinoma (HCC) is uniquely suited to transplantation: LT simultaneously removes the tumor and addresses the underlying liver disease—most commonly cirrhosis—and therefore represents the treatment of choice for HCC without extrahepatic spread in eligible patients [1]. Poor outcomes with recurrence rates of over 50% and 5-year survival rates of 20–40% in the 1980s led to a debate as to whether HCC as an indication for transplantation was justified [2]. Ultimately, the Milan criteria (one tumor <5 cm or up to three tumors < 3 cm each, no vascular invasion, no extrahepatic spread) were implemented in 1996, which still form the basis for the indication and allocation of liver transplants in HCC patients in several countries worldwide [3]. This improved post-transplant results in terms of recurrence, graft and patient survival significantly: A consensus conference report summarized that LT within the Milan criteria yields a 5-year survival rate of approximately 70% [4]. Currently, recurrence rates of 8–20% and 5-year survival rates of around 70% are described in the literature [5,6]. With HCC representing a growing percentage of liver transplantations, numerous other criteria such as University of California San Francisco (UCSF) criteria (a single lesion of ≤ 6.5 cm or 2–3 lesions ≤ 4.5 cm with a total diameter ≤ 8 cm) or “Up-to-7” (the sum of the size of the largest tumor in cm and the number of tumors ≤ 7) have been implemented in recent years with the aim of allowing patients with even more advanced disease compared to the Milan criteria to undergo transplantation [7,8]. These are already included as recommendations in the current German and international guidelines [5,9]. Even more aggressive approaches have been described in the literature such as the Extended Toronto Criteria, which offer transplantation to all HCC recipients as long as no extrahepatic spread, poorly differentiated tumors or cancer related symptoms are present [10]. What all classifications have in common is that a definitive assessment is only possible based on histopathological analysis of the explanted livers. In addition, BT may achieve downsizing and render patients eligible for transplantation [5]. Among available bridging therapies, locally ablative treatments such as radiofrequency ablation (RFA) and microwave ablation (MWA) are generally reserved for smaller tumors [11,12]. Another form of local ablation is percutaneous ethanol injection (PEI), which has been established the longest but largely replaced by thermal ablation due to inferior results [13]. Transarterial approaches include transarterial chemoembolization (TACE) (conventional and drug-eluting bead variants) and transarterial radioembolization/selective internal radiotherapy (TARE/SIRT); recent data suggest potential superiority of TARE over TACE in larger lesions of unresectable HCC [14]. Multiple predictive factors towards post-transplant survival are described in the literature such as AFP levels by Duvoux et al. [15]. Other evidence suggests the etiology of liver disease plays a key role in post-transplant recurrence with alcoholic liver disease and high AFP levels seemingly being an unfavorable combination [16]. In 2017, Mehta et al. published the RETREAT score and described AFP at transplantation, microvascular invasion, sum of largest viable tumor + number of viable tumor nodes as predictive factors for post-transplant HCC recurrence [17]. Mazzaferro et al. published the Metroticket 2.0 calculator to predict survival after LT for HCC based on preoperatively and postoperatively available variables (AFP, size of largest vital tumor and number of vital nodes vs. size of largest nodule and number of nodules in explant pathology) [18]. When recurrence occurs, management is usually limited to surgery, locoregional therapy and tyrosine kinase inhibitors (TKIs) due to the substantial risk of acute rejection following the application of checkpoint inhibitors, however successful application has been described in the literature [19,20]. The present study aimed to evaluate the impact of different pre-transplant bridging strategies on post-transplant outcomes and to identify clinicopathological risk factors for HCC recurrence following LT in a large single-center cohort.

## 2. Materials and Methods

### 2.1. Study Design

We retrospectively analyzed 1416 patients undergoing LT at the Department of General, Visceral and Transplant Surgery at Hannover Medical school between January 2007 and September 2022. Of these 185 were performed due to histopathologically confirmed HCC as indication for LT. Combined transplantations, retransplantation during the index hospitalization, pediatric transplantation in recipients under the age of eighteen and other reasons than HCC for LT were excluded from further analysis. The last day of follow-up was 17 April 2024, resulting in a mean observational period of 73 months.

### 2.2. Definition of Variables

For subgroup analysis the patient collective was further divided in two groups: without recurrence of HCC and with intra or extrahepatic recurrence of histopathologically confirmed HCC. The body mass index (BMI) was calculated, using the common formula, as weight (kilograms) divided by height (meters) squared. The glomerular filtration rate was calculated using the CKD-Epi formula [21]. The laboratory Model of End Stage Liver Disease (LabMELD) score was calculated using the formula first described by Kamath et al. [22]. The exceptional Model of End Stage Liver Disease (ExcMELD) score is based on the LabMELD score with additional points awarded every 3 months as an equivalent to a 10% increased waiting list mortality [23]. Early allograft dysfunction (EAD) was defined as the presence of either: ALT/AST > 2000 U/L within first 7 postoperative days, bilirubin ≥ 10 mg/dL on postoperative day 7 or INR ≥ 1.6 on postoperative day 7 in accordance with the definition published by our group before [24]. Extended criteria donor (ECD) organs were defined using Eurotransplant criteria when one of the following was met: donor age > 65 years, duration of mechanical ventilation prior to explantation > 7 days, BMI > 30, bilirubin > 3 mg/dL, aspartate aminotransferase (AST) > 105 U/L, alanine aminotransferase (ALT) > 90 U/L, sodium > 165 mmol/L, and hepatic steatosis > 40% [25]. Liver cirrhosis (micro and macronodular), as well as portal vein thrombosis, was histopathologically confirmed. Primary aortic anastomosis was defined as at least one vessel being directly anastomosed onto the recipient’s aorta. If one vessel of the donor was directly anastomosed to one vessel of the recipient (excluding the aorta), anastomoses were defined as “standard”. All other anastomoses were defined as “complex vessel reconstructions”, e.g., usage of additional vascular grafts. Milan, Up-to-7 and UCSF criteria were defined according to their original publications by Mazzafero and Yao et al. [3,7,8]. Metroticket 2.0 score was calculated using the Metroticket 2.0 online calculator with results from definitive postoperative pathology findings [18]. Pre-transplant HCC diagnosis was established based on dynamic contrast-enhanced imaging in accordance with contemporaneous EASL guidelines (LI-RADS criteria applied from 2018 onward), without routine percutaneous tumor biopsy, consistent with standard clinical practice. Histological tumor grade and vascular invasion were therefore assessed exclusively from explant pathology in all cases.

### 2.3. Statistical Analysis

Normal distribution was assessed using Kolmogorov–Smirnov test. Metric variables between the two groups were compared using Student’s *t*-test in case of normal distribution, otherwise the Mann–Whitney U-test was used. Categorical variables were compared using the Chi^2^ test or the Fisher-Exact test, respectively. For Kaplan–Meier analysis, the log-rank test was applied. When comparing more than two groups, the overall log-rank test was performed first. In case of a significant overall test, subsequent pairwise comparisons were conducted using the log-rank test with Holm–Šidák correction for multiple comparisons. Survival estimate for each group has been reported. For identification of independent risk factors regarding OS, multivariate Cox regression analysis was performed by incorporating a priori defined covariates based on clinical relevance, with missing values < 10%. Statistical significance was set at a *p* value of <0.05 and is shown in bold (tables). The data collected was analyzed using SPSS statistical software (SPSS version 30 for Mac; SPSS Inc.; IBM Corporation, Armonk, NY, USA) and GraphPad Prism (GraphPad Prism version 11.0.0 for Windows, GraphPad Software, Boston, MA, USA). Figures were created using GraphPad as well.

### 2.4. Ethics

Within the general policy of our institution, patients or their legal guardians provided informed consent that their data may be used for scientific purposes. The ethics committee at Hannover Medical School stated that no further approval for retrospective data collection is needed. Prior analysis, patient data and records were anonymized and de-identified.

## 3. Results

### 3.1. Recipient and Donor Characteristics and Bridging Therapy

The median recipient age was 59 years (range: 20–71 years), and 79.5% of patients were of a male gender (*n* = 147). Recipients had a median BMI of 27.0 kg/m^2^ (17.1–39.5 kg/m^2^). Viral hepatitis represented the most common underlying liver disease in 58.4% of cases (*n* = 108), followed by alcohol-associated liver disease (*n* = 29, 15.7%). Right before transplantation, recipients had a median LabMELD score of 10 (6–40), while the median ExcMELD was 28 (22–40). The median time on waitlist was 186 days (1–3462 days). A total of 85.4% of patients underwent prior bridging therapy including only TACE (*n* = 20, 10.8%), only local ablation (*n* = 32, 17.3%), only liver resection (*n* = 27, 14.6%) or a combination of different approaches (*n* = 50, 27%). Few patients received different and partially outdated forms of BT, such as PEI in 22.7% (*n* = 42) of cases, and *n* = 1 patient each received proton beam therapy, stereotactic body radiation therapy (SBRT), and downstaging after receiving palliative care treatment with tyrosine kinase inhibitors (TKIs). For further analysis, the decision was made to analyze patients receiving multiple forms of BT and less established BTs separately to increase discriminative ability between BTs used.

Donors were 56.8% male (*n* = 105) with a median age of 55 years (18–88 years) and a median BMI of 27.0 kg/m^2^ (13.8–59.0). ECD organs were transplanted in 65.4% of cases (*n* = 121). Donor stay in the Intensive Care Unit (ICU) prior to explantation was a median of 4 days (0.5–20.0 days). A total of 36 donors (19.5%) had been ventilated for more than 7 days prior to explantation.

No significant differences were found in demographic characteristics such as age, sex, BMI, or LabMELD scores comparing recurrence-free recipients (*n* = 168) with patients suffering post-transplant HCC recurrence (*n* = 17). However, the prevalence of viral hepatitis was higher in the recurrence group and the rate of alcohol-associated liver disease was correspondingly lower (70.6% vs. 57.1% with viral hepatitis and 0.0% vs. 17.3% with alcohol toxic liver disease, *p* = 0.01). Patients with post-transplant HCC recurrence had significantly higher peak AFP levels before transplantation (median 37.5 vs. 10 ng/mL, *p* = 0.03) as well as last measured AFP levels (median 21.5 vs. 6 ng/mL, *p* = 0.02). Furthermore, donors in the recurrence group were more frequently ventilated for over 7 days prior organ procurement (41.2% vs. 17.3%, *p* = 0.03) and had a higher BMI (30.14 vs. 27.44 kg/m^2^, *p* = 0.03), resulting in higher ECD rates, although the latter was not significant (82.4% vs. 63.7%, *p* > 0.05). Table 1 provides an overview of the comparative descriptive analysis of recurrence-free patients and recipients suffering post-transplant HCC recurrence.

Histopathological tumor burden was retrospectively assessed using several established classifications, which showed a major proportion of the cohort transplanted within the Milan (*n* = 105, 67.7%), University of California San Francisco (UCSF) (*n* = 122, 78.7%) and “Up-To-Seven” criteria (*n* = 131, 84.5%). No significant differences towards recurrence rates were found with respect to Milan, UCSF or “Up-To-Seven” criteria. In 32 recipients (17.2%) retrospective classification was not possible, and they were subsequently excluded from the above classifications.

### 3.2. Surgical Details, Histopathological Findings and Perioperative Outcome

A primary aortic anastomosis was performed in six cases (3.2%) and complex arterial reconstruction in 27 cases (14.6%). The median operative time was 220 min (114–573 min). Grafts underwent a median cold ischemia time of 586 min (313–1098 min) and a median warm ischemia time of 48 min (22–181 min). The median graft weight was 1759.5 g (833–3074 g). Intraoperative transfusion was required with a median of six units of packed red blood cells (PRBCs) (0–38), 10 units of fresh frozen plasmas (FFP) (0–35) and one unit of platelet concentrate (PC) (0–6). In 19 cases (10.3%) Primary Nonfunction (PNF) was observed with an EAD rate in our patient collective of 55.7% (*n* = 103). Retransplantation was required in 8.6% of patients (*n* = 16), most commonly due to PNF or vascular complications, with a median time to retransplantation of 4.5 days (1–735 days). The median ICU/ Intermediate Care Unit (IMC) stay was 9.5 days (3–161 days) with 20 patients (10.8%) dying during their ICU stay due to early postoperative complications.

In 95.1% of explanted livers, histopathological examination revealed liver cirrhosis (*n* = 176), most commonly of the micronodular type (*n* = 109, 58.9%). The average tumor size was 2.95 cm (0.4–9.5 cm), with a median of one tumor per patient (1–20). A tumor necrosis possibly reflective of a response to previous BT was seen in 76 patients (58.0%). In 59 patients (31.9%) histopathological examination revealed no proof of malignancy, which was interpreted as a complete response to prior BT. Most tumors were graded to moderately differentiated (G1 in eight cases (6.3%) and G2 in 88 cases (69.8%), G3 in nine (7.1%) cases. Tumor-free resection margins (R0) were achieved in all cases. Macrovascular invasion only occurred in one patient (0.8%). In 11.4% of cases (*n* = 21) portal vein thrombosis was observed.

Patients with HCC recurrence showed significantly larger tumor sizes compared to those recipients without (median 3.95 cm vs. 2.6 cm; *p* = 0.029). Portal vein thrombosis and vascular invasion (macro and microvascular) were also more common among recurrence patients, although this was not significant. Additionally, grade 3 tumors (G3) were notably more frequent in patients suffering post-transplant HCC recurrence (25.0% vs. 5.3%, *p* = 0.04). Furthermore, the HCC recurrence group showed significantly lower rates of micronodular liver cirrhosis (35.3% vs. 61.3%, *p* = 0.04) and consequently higher rates of macronodular liver cirrhosis (21.4% vs. 35.5%, *p* > 0.05). Table 1 gives an overview of the general cohort as well as of the comparative descriptive analysis of recurrence-free patients and recipients suffering post-transplant HCC recurrence.

The time to recurrence was highly variable with a median of 845 days (126–3978 days). Also, the locations of tumor recurrence were diverse including intrahepatic recurrence in 11 cases (64.7%), pulmonary recurrence in nine cases (52.9%), lymph nodes (29.4%), bone (29.4%), and others, such as chest or abdominal wall, adrenal glands, mediastinum and peritoneum. Median AFP peak level after HCC recurrence was 54 ng/mL (3–32,153 ng/mL). AFP negative recurrences occurred in *n* = 3 (17.6%) cases. The therapy of HCC recurrence consisted of mostly multimodal treatment approaches including surgical excision in eight cases (47.1%), radiotherapy (52.9%), SIRT (17.6%), and systemic therapies (such as Sorafenib or Lenvatinib in 70.6%). Consequently, we observed a significantly higher mortality in patients suffering post-transplant HCC recurrence during the observational period (88.2% vs. 39.9%, *p* < 0.01). Regarding treatment of HCC recurrence, patients receiving combined locoregional and systemic therapy showed numerically longer median survival compared to those treated with systemic therapy alone (median 13 vs. 2 months), though formal statistical testing was precluded by the small recurrence cohort (*n* = 17). Table 2 gives further insights into characteristics of patients suffering HCC recurrence after LT.

### 3.3. Analysis of Overall and Disease-Free Survival

**Figure 1:**
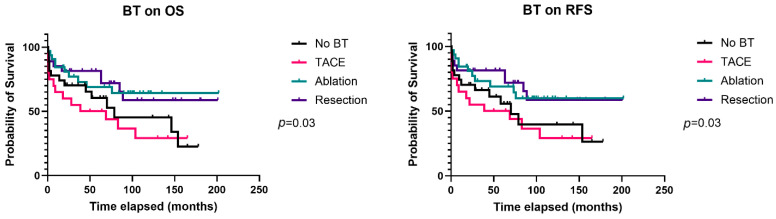
Comparative Kaplan–Meier Survival analysis for overall patient survival and recurrence-free survival stratified regarding BT received prior to LT after excluding patients who received different forms of BT and combination treatments (*n* = 106 remaining). Log-rank test was used for survival comparison among the groups.

Analysis of OS revealed significant overall differences among bridging therapy groups (overall log-rank *p* = 0.03). Numerically, recipients who had undergone local ablation or surgical resection prior to LT showed improved OS compared to those receiving TACE or no bridging therapy; however, none of the pairwise comparisons reached statistical significance after Holm–Šidák correction for multiple testing. Similarly, the overall log-rank test for RFS showed significant differences among groups (*p* = 0.03), with the same numerical trend favoring ablation and resection, but again without significant pairwise differences after correction (Figure 1).

**Figure 2:**
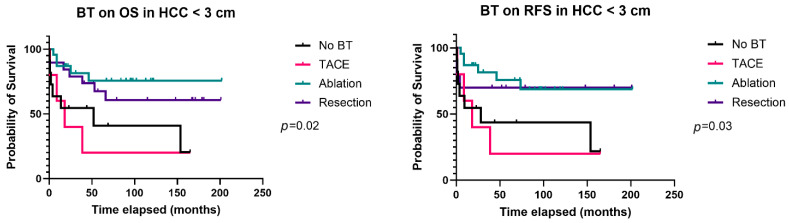
Comparative Kaplan–Meier Survival analysis for overall patient survival and recurrence-free survival stratified regarding BT received prior to LT after excluding patients who received different forms of BT and combination treatments in patients with early HCC < 3 cm (*n* = 49 remaining). Log-rank test was used for survival comparison among the groups.

Subgroup analysis of patients with early HCC < 3 cm (*n* = 81) included no BT in *n* = 11 cases (13.6%), combination in *n* = 23 cases (28.4%), TACE in *n* = 5 (6.2%), local ablation in *n* = 23 (28.4%), resection in *n* = 10 (12.4%), and PEI in *n* = 9 (11.1%). In this subgroup, pairwise comparison after Holm–Šidák correction demonstrated significantly improved OS for local ablation compared to TACE (*p* = 0.035). A similar trend was observed for RFS, although this did not reach statistical significance after correction (*p* = 0.058). No other pairwise comparison reached significance in this subgroup (Figure 2).

**Figure 3:**
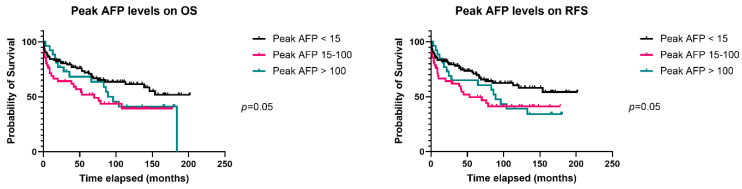

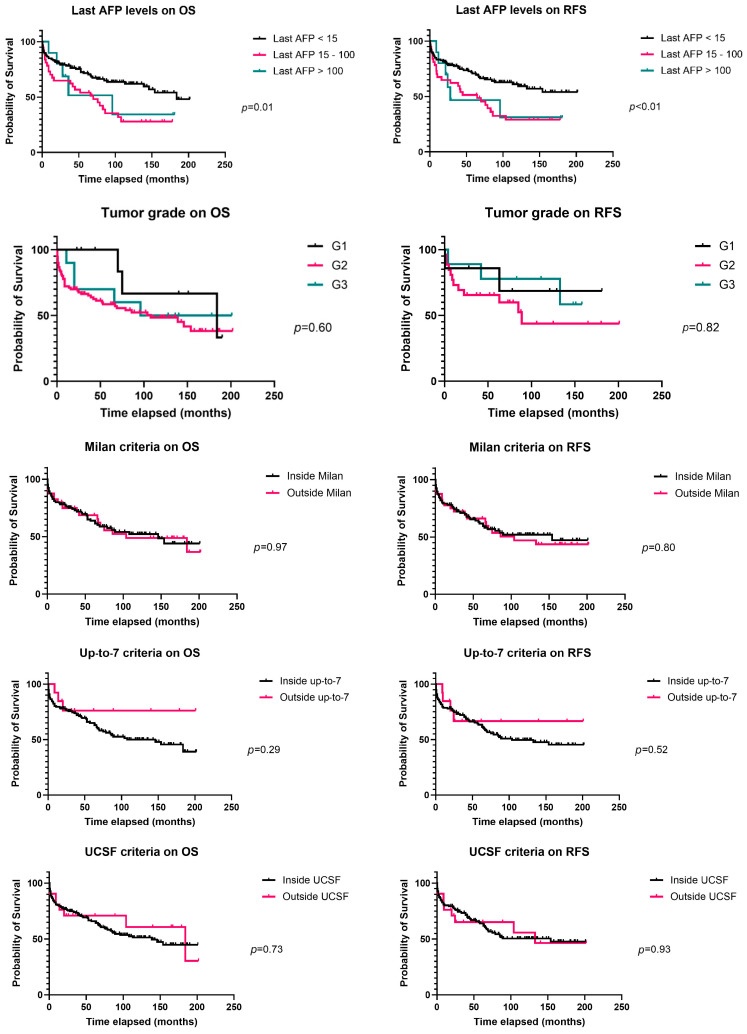
Comparative Kaplan–Meier Survival analysis for Milan, Up-to-7 and UCSF criteria and their effect on OS and RFS and peak/last AFP levels divided into categories of <15 ng/mL, 15–100 ng/mL and >100 ng/mL and their effect on OS and RFS as well as tumor grading (G1, G2, G3) and its effect on OS and RFS. Log-rank test was used for survival comparison among the groups.

Using Kaplan–Meier analysis, we were further able to show that established listing criteria such as Milan, Up-to-7 and UCSF criteria do not significantly correlate with OS in our cohort. Beyond tumor size-based criteria, tumor biology represented by AFP levels were also evaluated based on the effect of peak and last measured AFP divided into subgroups of <15 ng/mL, 15–100 ng/mL, >100 ng/mL, which showed significant overall differences. After Holm–Šidák correction for pairwise comparisons, the last measured pre-transplant AFP < 15 ng/mL was associated with significantly improved OS (*p* = 0.006) and RFS (*p* = 0.008) compared to the 15–100 ng/mL group. In contrast, peak AFP did not show significant pairwise differences after correction. No significant pairwise differences were observed for tumor grading (G1, G2, G3) regarding OS or RFS after correction for multiple comparisons. Details are shown in Figure 3.

Multivariable Cox proportional hazards regression, incorporating clinically preselected variables, identified HCC recurrence after LT, recipient BMI, and recipient LabMELD score as independent regarding OS during the observational period. Further details are shown in Table 3.

## 4. Discussion

Liver transplantation for HCC was first systematically evaluated in 1996 by Mazzaferro et al., who established the Milan criteria (single nodule ≤ 5 cm or up to three nodules ≤ 3 cm each) as the benchmark for LT candidacy. Size thresholds were chosen as surrogate parameters for favorable tumor biology and low recurrence risk, yielding a recurrence rate of 17% after a median follow-up of 26 months [3]. Since then, listing criteria have been progressively expanded beyond Milan whilst maintaining acceptable recurrence rates. In the present cohort, a recurrence rate of 9% over an observational period of up to 15 years was observed, consistent with recent meta-analyses of Western transplant populations [26]. The proportion of LT performed for HCC in the present cohort (13%) is lower than in some international series, which reflects the organ allocation framework of Eurotransplant, the conservative application of extended listing criteria during the early study period (2007–2014), and the higher proportion of non-oncological indications especially due to a high number of pediatric transplantations at our center. A relative increase in HCC listing in more recent years is consistent with the broader European trend toward tumor-biology-guided allocation policies. The underlying etiology of liver disease significantly influenced recurrence risk: approximately 70% of recurrences occurred in patients with viral hepatitis, while no recurrences were observed among patients with alcohol-associated liver disease. This is consistent with published data describing Hepatitis-B-positive recipients as a high-risk group, whereas recurrence risk in alcohol-associated cirrhosis appears particularly driven by elevated peak AFP levels [16]. The implementation of bridging locoregional therapies was motivated primarily by reducing waitlist dropout rather than recurrence risk, and their use is recommended in current German guidelines [9,27]. International guidelines recommend choice of BT based on individual selection from available options such as TACE, SIRT, local ablation, resection and SBRT [28]. In the present cohort, the overall log-rank test showed significant differences in OS and RFS among bridging therapy groups (each *p* = 0.03), with numerically superior outcomes for ablation and resection compared to TACE or no bridging therapy; however, pairwise comparisons did not reach significance after Holm–Šidák correction in the overall cohort, likely attributable to small subgroup sizes. Notably, in the early HCC subgroup (<3 cm), local ablation was associated with significantly improved OS compared to TACE after correction (*p* = 0.035), with a similar trend for RFS (*p* = 0.058), which may suggest a curative potential for local ablation in early-stage disease, although the small subgroup sizes warrant cautious interpretation. [29]. We observed similar non-statistically significant recurrence rates when comparing MWA or RFA to PEI. We observed a higher mean waiting list time in the HCC recurrence group (316 vs. 386 days); however, no statistical significance was reached, possibly due to the small sample size in our cohort. There is substantial evidence that waiting time before LT for HCC has a benefit in selecting for patients via a “test of time” with aggressive tumor biologies who progress outside of allocation policies due to extrahepatic spread or vascular invasion during their time on the wait list [30,31]. No “ideal waiting time” has been established to date. A numerically higher mean donor BMI was observed in the recurrence subgroup, consistent with prior reports linking visceral adiposity and graft steatosis to oncogenic signaling and ischemia–reperfusion injury [32,33]. We also observed a non-significant trend towards higher recurrence rates in recipients of ECD organs, potentially reflective of increased graft inflammation and ischemia–reperfusion injury [34]. Beyond reducing waitlist dropout, locoregional and systemic treatment for intermediate and advanced stage HCC can achieve downstaging to within established transplant criteria [35]. While we observed significantly larger tumors in the HCC recurrence subgroup (3.62 cm vs. 2.85 cm; *p* = 0.029), no differences were observed with regards to patients being inside or outside of Milan, UCSF and Up-to-7 criteria (recurrence rate and via Kaplan–Meier curves), which is in line with a recent transplant registry study with over 25.000 recipients showing that outcomes are comparable between the allocation models [36]. Sapisochin et al. were able to show that AFP levels > 500 ng/mL are associated with an increased risk of recurrence in patients with HCC [10]. Although we could not observe the cut-off of 500 ng/mL in our patient collective, we confirmed a significantly higher median peak and last recorded AFP in our recurrence subgroup (e.g., median peak AFP 37.5 vs. 10 ng/mL; *p* = 0.02). Many systems have been developed over the years for guiding liver transplantation candidacy that include AFP values such as the French “AFP model” and the United Network for Organ Sharing “Downstaging criteria”, which are in theory supported by our findings [15,37]. AFP correlates well with vascular invasion but its sensitivity in detecting HCC recurrence varies and, similar to our experience of 20% AFP-negative recurrences, underestimation of recurrence risk is possible. Most explant pathologies showed moderately differentiated HCC (69.8%), although we saw a significantly increased risk of recurrence in the poorly differentiated tumors (5.3% vs. 25%; *p* = 0.04), supporting the Extended Toronto Criteria by Sapisochin et al. [10]. Across different allocation criteria, consensus exists that macrovascular invasion is associated with an unacceptable safety profile when downstaging is unsuccessful; however, if successful, outcomes are similar to patients never showing macrovascular invasion [38]. Out of the 17 patients (9.2%) that suffered recurrence, median time from transplant to diagnosis of recurrence was around 2.5 years with considerable variance ranging from only 4 months to 11 years. A total of 17.4% of patients experienced AFP-negative recurrence. Many authors differentiate between early (<2 years after LT) and late HCC recurrence with early recurrence being associated with poor prognosis and early death, which is in line with our observations (7 months vs. 22 months median OS) [39]. Possible mechanisms for early recurrence are micrometastases in other organs that are missed during pretransplantation workup and grow rapidly under immunosuppression but might also be associated with circulating tumor cells that engraft in the new organ after liver transplantation [40]. Site of recurrence was predominantly the liver (64.7%), which is high compared to other published reports, but also the lungs, lymph nodes, bone and peritoneal and pleural metastasis, which are well described in the literature [41,42]. In keeping with recent data reporting intrahepatic recurrence as an adverse prognostic factor in post-LT HCC, patients with intrahepatic recurrence in our cohort showed a numerically shorter median OS compared to those with extrahepatic recurrence (13 vs. 19 months), although formal statistical testing was precluded by the small subgroup size [43]. Almost 50% of our patients were amenable to surgical resection of their HCC recurrence, which is also high compared to the literature but likely due to the high rate of intrahepatic recurrence. The most common form of treatment was systemic treatment with TKIs at some point during disease progression (70.6% of cases), which is also in line with previously published results [39,41]. TKIs have been proven to be a valuable choice in the treatment of recurrent HCC [44]. The use of ICIs has been traditionally avoided due to the fear of rejection and evidence is still scarce; however, some studies suggest an acceptable safety profile and promising results [45]. Prognosis after HCC recurrence is poor with 5-year OS as low as 13.3%, similar to a recent registry report [46]. Interestingly, around 5% of patients in our collective also developed de novo different malignant diseases after LT, which is likely aggravated by immunosuppression [47]. In a multivariate Cox regression model, HCC recurrence was an independent predictor of reduced OS after LT, which is well described in the literature [39]. Another independent risk factor was recipient BMI, which did not differ significantly between the two groups observed; however, we were able to show corresponding significant differences in donor BMI. Underlying mechanisms possibly also include the oncogenic potential of visceral adipose tissue and increased acquired graft steatosis facilitating inflammation in the graft [32,33,34]. Interestingly, we were able to show low recipient LabMELD as independent risk factor for reduced OS during the observational period, which has not been described in the literature thus far. One possible, albeit speculative, explanation is that low LabMELD may be reflective of a high oncologic burden of disease, with a high LabMELD being associated with lower oncologic risk and more decompensated cirrhosis. Considering the high mortality of HCC recurrence in patients with high oncologic risk, HCC recurrence might influence prognosis more than advanced cirrhosis in an HCC-only collective. This interpretation aligns with the allocation reality of Eurotransplant, where HCC patients receive exceptional MELD points based on oncologic urgency rather than hepatic decompensation. Accordingly, recipients with a low LabMELD represent a subgroup in whom tumor biology dominates the prognostic trajectory. The paradoxical inverse relationship between LabMELD and OS in this HCC-exclusive cohort therefore reflects the hypothesis that oncologic risk, not hepatic reserve, might be the primary determinant of long-term outcome in this indication.

The limitations of our study lie in its retrospective, single-center design and the inclusion of a heterogenous patient cohort, which partially resulted in small group sizes.

Overall, our findings show that the use of pretransplant bridging therapies via ablation correlates with an improved OS and RFS in small HCCs under 3 cm. To further support these findings prospective clinical trials, multicenter studies and meta-analyses are required. Furthermore, the expansion of allocation policies beyond the Milan criteria and towards criteria focused on tumor biology and response to bridging therapies rather than pure size-based considerations may help reduce the incidence of HCC recurrence while safely expanding access to LT for more patients.

## Figures and Tables

**Table 1:** Overview of recipient and donor characteristics (*n* = 185) and comparative descriptive analysis of recurrence-free patients (*n* = 168) and recipients suffering post-transplant HCC recurrence (*n* = 17); for more details regarding donor and recipient data, operative details, outcome parameters and histopathological analysis, see Table S1 in the Supplements.

Variables			Overall	Without Recurrence	Recurrence After LT	Missing Value (%)	*p* Value
Baseline recipient data	Age (y)		56.91; 59 (20–71)	56.95; 59 (20–71)	56.47; 59 (41–68)	1 (0.5)	0.747
	Body mass index (kg/m^2^)		27.45; 27 (17.7–39.5)	27.43; 26.95 (17.7–39.5)	27.69; 27.2 (22.6–35.8)	0 (0)	0.814
	LabMELD		13.56; 10 (6–40)	13.79; 10 (6–40)	11.24; 10 (6–31)	0 (0)	0.234
	ExcMELD		27.23; 28 (22–40)	27.31; 28 (22–40)	26.36; 26.5 (22–31)	23 (12.4)	0.426
	Time on wait list (d)		322.89; 186 (1–3462)	316.47; 185.5 (1–3462)	386.29; 211 (8–3015)	0 (0)	0.671
Donor data	Age (y)		54.86; 55 (18–88)	54.57; 55 (18–88)	57.76; 61 (29–78)	0 (0)	0.292
	Body mass index (kg/m^2^)		27.69; 27 (13.8–59.0)	27.44; 26.7 (13.8–59.0)	30.14; 29.4 (24.2–42.3)	0 (0)	**0.027**
	Extended criteria donor (ECD)		121 (65.4)	107 (63.7)	14 (82.4)	0 (0)	0.181
Tumor marker	First AFP pre-transplantation (ng/mL)		3871.11; 7.0 (1.0–640,886.0)	224.74; 7 (1–30,330)	40,334.88; 10.0 (2.0–640,886.0)	9 (4.9)	0.372
	Peak AFP pre-transplantation (ng/mL)		6266.72; 10.0 (1.0–975,635.0)	614.1; 10 (1–56,100)	61,379.81; 37.5 (2.0–975,635.0)	13 (7.0)	**0.027**
	Last AFP pre-transplantation (ng/mL)		359.97; 6.0 (1.0–56,100.0)	376.1; 6 (1–56,100)	200.69; 21.5 (2.0–2572.0)	11 (5.9)	**0.020**
Bridging therapy	Overall		158 (85.4)	145 (87.4)	13 (76.5)	0 (0)	0.221
	Surgical resection		44 (23.8)	40 (23.8)	4 (23.5)		1.000
	Percutaneous ethanol injection (PEI)		42 (22.7)	38 (22.6)	4 (23.5)		1.000
	Microwave ablation (MWA)		16 (8.6)	15 (8.9)	1 (5.9)		1.000
	Radiofrequency ablation (RFA)		48 (25.9)	44 (26.2)	4 (23.5)		1.000
	Tyrosine kinase inhibitors (TKIs)		1 (0.5)	1 (0.6)	0 (0)		1.000
	Stereotactic body radiation therapy (SBRT)		1 (0.5)	1 (0.6)	0 (0)		1.000
	Unspecified local ablation		1 (0.5)	1 (0.6)	0 (0)		1.000
	Selective internal radiotherapy (SIRT)		1 (0.5)	0 (0)	1 (5.9)		0.092
	Proton beam therapy		1 (0.5)	1 (0.6)	0 (0)		1.000
	Transarterial chemoembolization (TACE)	Overall	51 (27.6)	47 (28.0)	4 (23.5)		0.784
		Number of TACE	1.73; 1.0 (1–6)	1.77; 1 (1–6)	1.25; 1 (1–2)		0.447
	Positive response to bridging therapy		71 (44.7)	64 (43.8)	7 (53.8)	2 (1.3)	0.570
Combination or single BT	Combination of different BT		50 (27)	44 (26.1)	6 (33.3)	0 (0)	0.293
	Only TACE		20 (10.8)	19 (11.3)	1 (5.5)		0.427
	Only resection		27 (14.6)	26 (15.5)	1 (5.5)		0.253
	Only local ablation (RFA/MWA)		32 (17.3)	30 (17.9)	2 (11)		0.407
	Only PEI		28 (15.1)	25 (14.9)	3 (16.5)		0.492
	No bridging therapy		27 (14.6)	23 (13.7)	4 (22.2)		0.221
Classifications	Inside Milan criteria		105 (67.7)	95 (68.3)	10 (71.4)	10 (6.5)	1.000
	Inside Up-to-7 criteria		131 (84.5)	120 (86.3)	11 (78.6)	11 (7.1)	0.332
	Metroticket predicted 5-year OS (%)		71.15; 73.05 (44.0–79.1)	71.56; 73.2 (44.0–79.1)	67.07; 71.9 (46.1–77.8)	11 (7.1)	0.203
	Inside UCSF criteria		122 (78.7)	112 (80.6)	10 (71.4)	12 (7.7)	0.407
	Retrospective classification for inside/outside criteria not possible		32 (17.2)	29 (15.6)	3 (17.6)	0 (0)	0.968
Outcome	Hemodialysis at discharge		3 (1.6)	3 (1.8)	0 (0)	1 (0.5)	1.000
	Time on IMC/ICU		18.17; 9.5 (3–161)	18.87; 10 (3–161)	12; 9 (3–33)	19 (10.3)	0.755
	Death on IMC/ICU		20 (10.8)	20 (11.9)	0 (0)	0 (0)	0.224
	Early allograft dysfunction (EAD)		103 (55.7)	95 (56.5)	8 (47.1)	5 (2.7)	0.442
	Retransplantation		16 (8.6)	15 (8.9)	1 (5.9)	0 (0)	1.000
	Death during observational period		82 (44.4)	67 (39.9)	15 (88.2)	0 (0)	**<0.01**
Histopathological examination of explanted liver	Portal vein thrombosis		21 (11.4)	17 (10.4)	4 (23.5)	18 (9.7)	0.062
	Liver cirrhosis	Overall	176 (95.1)	163 (97.0)	13 (76.5)	7 (3.8)	0.152
		Micronodular	109 (58.9)	103 (61.3)	6 (35.3)	57 (30.8)	**0.041**
		Macronodular	42 (22.7)	36 (21.4)	6 (35.3)		0.079
	Number of tumors		1.94; 1 (1–20)	1.87; 2 (1–8)	2.91; 1 (1–20)	8 (6.1)	0.123
	Tumor size (cm)		2.95; 2.85 (0.4–9.5)	2.85; 2.6 (0.4–9.5)	3.62; 3.95 (1.2–5.3)	8 (6.1)	**0.029**
	Tumor necrosis present		76 (58.0)	64 (56.1)	9 (75.0)	6 (4.6)	0.197
	Vascular invasion	Overall	13 (10.5)	10 (8.8)	3 (17.6)	2 (1.6)	0.091
		Microvascular	11 (8.9)	9 (8.0)	2 (11.8)	3 (2.4)	0.256
		Macrovascular	1 (0.8)	0 (0)	1 (5.9)	4 (3.2)	0.090
	T status	T1	72 (57.1)	65 (57.0)	7 (58.3)	0 (0)	1.000
		T2	48 (38.1)	45 (39.5)	3 (25.0)		0.533
		T3	5 (4.0)	3 (2.6)	2 (16.7)		0.071
		T4	1 (0.8)	1 (0.9)	0 (0)		1.000
	N status	N0	66 (52.4)	60 (52.6)	6 (50.0)	0 (0)	1.000
		N1	1 (0.8)	1 (0.9)	0 (0)		1.000
	G Status	G1	8 (6.3)	7 (6.1)	1 (8.3)	21 (16.7)	0.562
		G2	88 (69.8)	81 (71.1)	7 (58.3)		0.509
		G3	9 (7.1)	6 (5.3)	3 (25.0)		**0.040**
	Resection margins	R0	126 (100)	114 (100)	12 (100)	0 (0)	n.a.
	L status	L0	61 (48.4)	57 (50.0)	4 (33.3)	65 (51.6)	0.367
	V status	V0	116 (92.1)	107 (93.9)	9 (75.0)	0 (0)	0.054
		V1	9 (7.1)	7 (6.1)	2 (16.7)		0.205
		V2	1 (0.8)	0 (0)	1 (8.3)		0.095

**Table 2:** Overview of recipients suffering HCC recurrence within the observed time frame (*n* = 17).

Variables		Recurrence After LT	Missing Value
Overall		17 (100)	0 (0)
Time after LT (d)		1236; 845 (126–3978)	0 (0)
Early recurrence (<2 years)		7 (41.2)	0 (0)
Late recurrence (>2 years)		10 (58.8)	0 (0)
Tumor size at diagnosis (cm)		2.46; 2.7 (0.5–4.2)	7 (41.2)
Location of HCC recurrence	Liver	11 (64.7)	0 (0)
	Lung	9 (52.9)	1 (5.9)
	Lymph nodes	5 (29.4)	
	Bone	5 (29.4)	
	Intestine	1 (5.9)	
	Mediastinum	1 (5.9)	
	Oral cavity	1 (5.9)	
	Spleen	2 (11.8)	
	Chest abdominal wall	7 (41.2)	
	Peritoneum	3 (17.6)	
	Soft tissue	3 (17.6)	
	Adrenal gland	1 (5.9)	
Different malignant disease after LT		0 (0)	0 (0)
Therapy for HCC recurrence	Surgical Excision	8 (47.1)	0 (0)
	MWA	1 (5.9)	
	SIRT	3 (17.6)	
	TACE	3 (17.6)	
	Embolization	1 (5.9)	
	Radiotherapy	9 (52.9)	
	Chemotherapy	1 (5.9)	
	Tyrosine kinase inhibitors	12 (70.6)	
	Systemic therapy combined with locoregional therapy	9 (52.9)	
AFP peak after recurrence		2844.33; 54 (3–32,153)	2 (11.8)
AFP negative recurrence		3 (17.6%)	2 (11.8)
Survival after HCC recurrence (months)	Overall	23.2; 13 (1–88)	2 (11.8)
	Early recurrence (<2 years)	10.28; 7 (1–31)	0 (0)
	Late recurrence (>2 years)	34.50; 22 (2–88)	2 (20)
	Systemic therapy only	3.33; 2 (1–7)	0 (0)
	Systemic therapy + locoregional therapy	17.42; 13 (7–53)	2 (11.8)
	Intrahepatic recurrence	19.72; 13 (1–68)	0 (0)
	Extrahepatic recurrence	32.75; 19 (5–88)	2
1-year overall survival		53.3%	2 (11.8)
2-year overall survival		26.7%	2 (11.8)
5-year overall survival		13.3%	2 (11.8)
1-month		100%	0 (0)
1-year		94.1%	
3-year		58.2%	
5-year		38.8%	
10-year		25.9%	
1-month		94.1%	0 (0)
1-year		88.2%	
3-year		52.3%	
5-year		39.2%	
10-year		26.1%	

**Table 3:** Multivariable Cox proportional hazards regression model for OS during the observational period (*n* = 82; 44.4% of recipients).

Variable	Exp (B)	Confidence Interval (95% CI)	*p* Value
HCC recurrence (yes)	2.43	[1.05–5.63]	**0.04**
Tumor size in explant pathology (cm)	1.04	[0.83–1.31]	0.69
Peak AFP level (ng/mL)	1	[1–1]	0.70
Last AFP level (ng/mL)	1	[1–1]	0.70
Extended criteria donor (yes)	0.94	[0.48–1.84]	0.88
Early allograft dysfunction (yes)	1.98	[0.10–3.96]	0.06
Recipient age (years)	1.01	[0.97–1.05]	0.88
Recipient BMI (kg/m^2^)	1.08	[1.01–1.15]	**0.03**
Cold ischemic time (min)	1.01	[0.99–1]	0.50
Graft weight (g)	0.99	[0.99–1]	0.18
Wait time (days)	0.99	[0.99–1]	0.13
Recipient LabMELD	0.95	[0.90–0.99]	**0.02**

## Data Availability

The raw data analyzed during the current study are available from the corresponding author on reasonable request.

## Supplementary Materials

The following supporting information can be downloaded at: https://www.mdpi.com/article/10.3390/jcm15124464/s1, Table S1: Overview of all recipient and donor data, operative details, outcome parameters and histopathological results.

## Abbreviations

The following abbreviations are used in this manuscript:

BMI	Body mass index
BT	Bridging therapy
Cm	Centimeter
TACE	Transarterial chemoembolization
TARE	Transarterial radioembolization
EAD	Early allograft dysfunction
ECD	Extended criteria donor
FFP	Fresh frozen plasma
HCC	Hepatocellular carcinoma
ICU	Intensive care unit
IMC	Intermediate care unit
LT	Liver transplantation
MWA	Microwave ablation
PC	Platelet concentrate
PNF	Primary nonfunction
PRBC	Packed red blood cells
RFA	Radiofrequency ablation
RFS	Recurrence-free survival
SBRT	Stereotactic body radiation therapy
SIRT	Selective internal radiotherapy
TKI	Tyrosine kinase inhibitors
UCSF	University of California San Francisco
