# Supplementary material for: Post-Transplant HCC Recurrence and Survival: Impact of Bridging Therapy and Tumor Biology in 185 Consecutive Liver Transplants
Source: J Clin Med. 2026 Jun 9;15(12):4464. doi: 10.3390/jcm15124464 (PMC13300792; doi:10.3390/jcm15124464)
Supplement: Supplementary file 1 [file jcm-15-04464-s001.zip › jcm-4323164-supplementary.pdf]

Table S1. Overview of all recipient and donor data, operative details, outcome parameters and histopathological results.

Variables			Overall	Without Recurrence	Recurrence after LT	Missing value (%)	P value
Baseline recipient data	Age (y)		56.91; 59 (20-71)	56.95; 59 (20-71)	56.47; 59 (41-68)	1 (0.5)	0.747
	Male sex		147 (79.5)	134 (79.8)	13 (76.5)	0 (0)	0.755
	Body mass index (kg/m²)		27.45; 27 (17.7-39.5)	27.43; 26.95 (17.7-39.5)	27.69; 27.2 (22.6-35.8)	0 (0)	0.814
	Retransplantation		5 (2.7)	5 (3.0)	0 (0)	1 (0.5)	1.000
	Underlying disease	Viral hepatitis	108 (58.4)	96 (57.1)	12 (70.6)	0 (0)	**0.010**
		Alcoholic liver disease	29 (15.7)	29 (17.3)	0 (0)		
		Cryptogenic cirrhosis	20 (10.8)	18 (10.7)	2 (11.8)		
		Metabolic disorder	8 (4.3)	8 (4.8)	0 (0)		
		Non-alcoholic fatty liver disease	13 (7.0)	13 (7.7)	0 (0)		
		Other	7 (3.8)	4 (2.4)	3 (17.6)		
	LabMELD		13.56; 10 (6-40)	13.79; 10 (6-40)	11.24; 10 (6-31)	0 (0)	0.234
	ExcMELD		27.23; 28 (22-40)	27.31; 28 (22-40)	26.36; 26.5 (22-31)	23 (12.4)	0.426
	Time on wait list (d)		322.89; 186 (1-3462)	316.47; 185.5 (1-3462)	386.29; 211 (8-3015)	0 (0)	0.671
Donor data	Age (y)		54.86; 55 (18-88)	54.57; 55 (18-88)	57.76; 61 (29-78)	0 (0)	0.292
	Male sex		105 (56.8)	96 (57.1)	9 (52.9)	0 (0)	0.800
	Body mass index (kg/m²)		27.69; 27 (13.8-59.0)	27.44; 26.7 (13.8-59.0)	30.14; 29.4 (24.2-42.3)	0 (0)	**0.027**
	Extended criteria donor (ECD)		121 (65.4)	107 (63.7)	14 (82.4)	0 (0)	0.181
	ECD criteria	Serum sodium > 165 mmol/l	6 (3.2)	6 (3.6)	0 (0)	0 (0)	1.000
		Serum AST > 105 U/l	41 (22.2)	37 (22.0)	4 (23.5)	0 (0)	0.142
		Serum ALT > 90 U/l	25 (13.5)	24 (14.3)	1 (5.9)	1 (0.5)	0.476
		Serum bilirubin > 3 mg/dl	1 (0.5)	1 (0.6)	0 (0)	2 (1.1)	1.000
		BMI > 30 kg/m²	41 (22.2)	35 (20.8)	6 (35.3)	0 (0)	0.217
		Ventilation > 7 d	36 (19.5)	29 (17.3)	7 (41.2)	0 (0)	**0.026**
		Age > 65 y	42 (22.7)	36 (21.4)	6 (35.3)	0 (0)	0.225
		Liver steatosis > 40 %	6 (3.2)	6 (3.6)	1 (5.9)	15 (8.1)	1.000
	Donor LabMELD		10.19; 9 (5-25)	10.2; 9 (5-25)	10.0; 9 (7-16)	18 (9.7)	0.498
	Hemodialysis in the last 7 days		2 (1.1)	2 (1.2)	0 (0)	0 (0)	1.000
	Time on ICU (d)		5.17; 4.0 (0.5-20.0)	5.04; 4 (0.5-20.0)	6.41; 7 (1-18)	1 (0.5)	0.260
	Time on ventilator (d)		5.0; 4.0 (0.5-20.0)	4.86; 4.0 (0.5-20.0)	6.41; 7.0 (1.0-18.0)	1 (0.5)	0.199
Tumor marker	First AFP pre-transplantation (ng/ml)		3871.11; 7.0 (1.0-640886.0)	224.74; 7 (1-30330)	40334.88; 10.0 (2.0-640886.0)	9 (4.9)	0.372
	Peak AFP pre-transplantation (ng/ml)		6266.72; 10.0 (1.0-975635.0)	614.1; 10 (1-56100)	61379.81; 37.5 (2.0-975635.0)	13 (7.0)	**0.027**
	Last AFP pre-transplantation (ng/ml)		359.97; 6.0 (1.0-56100.0)	376.1; 6 (1-56100)	200.69; 21.5 (2.0-2572.0)	11 (5.9)	**0.020**
Bridging therapy	Overall		158 (85.4)	145 (87.4)	13 (76.5)	0 (0)	0.221
	Surgical resection		44 (23.8)	40 (23.8)	4 (23.5)		1.000
	Percutaneous ethanol injection (PEI)		42 (22.7)	38 (22.6)	4 (23.5)		1.000
	Microwave ablation (MWA)		16 (8.6)	15 (8.9)	1 (5.9)		1.000
	Radiofrequency ablation (RFA)		48 (25.9)	44 (26.2)	4 (23.5)		1.000
	Tyrosine kinase inhibitors (TKI)		1 (0.5)	1 (0.6)	0 (0)		1.000
	Stereotactic body radiation therapy (SBRT)		1 (0.5)	1 (0.6)	0 (0)		1.000
	Unspecified local ablation		1 (0.5)	1 (0.6)	0 (0)		1.000
	Selective internal radiotherapy (SIRT)		1 (0.5)	0 (0)	1 (5.9)		0.092
	Proton beam therapy		1 (0.5)	1 (0.6)	0 (0)		1.000

	Transarterial chemoembolization (TACE)	Overall	51 (27.6)	47 (28.0)	4 (23.5)		0.784
		Number of TACE	1.73; 1.0 (1-6)	1.77; 1 (1-6)	1.25; 1 (1-2)		0.447
	Positive response to bridging therapy		71 (44.7)	64 (43.8)	7 (53.8)	2 (1.3)	0.570
Combination or single BT	Combination of different BT		50 (27)	44 (26.1)	6 (33.3)	0 (0)	0.293
	Only TACE		20 (10.8)	19 (11.3)	1 (5.5)		0.427
	Only resection		27 (14.6)	26 (15.5)	1 (5.5)		0.253
	Only local ablation (RFA / MWA)		32 (17.3)	30 (17.9)	2 (11)		0.407
	Only PEI		28 (15.1)	25 (14.9)	3 (16.5)		0.492
	No bridging therapy		27 (14.6)	23 (13.7)	4 (22.2)		0.221
Initial immunosuppression	Ciclosporin based		52 (28.1)	44 (31.2%)	8 (47.1)	27 (14.6)	0.149
	Tacrolimus based		104 (56.2)	95 (67.9%)	9 (56.3)	29 (15.7)	0.253
	mTor inhibitor based		9 (4.9)	8 (5.7)	1 (5.9)	27 (14.6)	0.651
	Mycophenolate mofetile based		130 (70.3)	120 (85.7)	10 (66.7)	30 (16.2)	0.070
	Steroid based		129 (69.7)	116 (82.9)	13 (81.3)	29 (15.7)	0.549
Classifications	Milan criteria	Inside Milan criteria	105 (67.7)	95 (68.3)	10 (71.4)	10 (6.5)	1.000
		Tumor size inside Milan	115 (74.2)	103 (74.1)	12 (85.7)	9 (5.8)	0.735
		Number of tumors inside Milan	132 (85.2)	120 (86.3)	12 (85.7)	10 (6.5)	1.000
		Macrovascular invasion (no)	149 (96.1)	136 (97.8)	13 (92.9)	4 (2.6)	0.177
	Up to seven criteria (“upto7”)	Inside up to 7 criteria	131 (84.5)	120 (86.3)	11 (78.6)	11 (7.1)	0.332
		Tumor size inside up to 7	82 (52.9)	73 (52.5)	9 (64.3)	11 (7.1)	0.395
		Combined tumor size inside up to 7	50 (32.3)	47 (33.8)	3 (21.4)	11 (7.1)	0.543
		Macrovascular invasion (no)	150 (96.8)	137 (98.6)	13 (92.9)	4 (2.6)	0.093
	Metroticket predicted 5-year OS (%)		71.15; 73.05 (44.0-79.1)	71.56; 73.2 (44.0-79.1)	67.07; 71.9 (46.1-77.8)	11 (7.1)	0.203
	UCSF criteria	Inside UCSF criteria	122 (78.7)	112 (80.6)	10 (71.4)	12 (7.7)	0.407
		Tumor size inside UCSF	87 (56.1)	76 (54.7)	11 (78.6)	31 (20.0)	**0.033**
		Number of tumors inside UCSF	36 (23.2)	36 (25.9)	0 (0)	13 (8.4)	**0.039**
		Macrovascular invasion (no)	147 (94.8)	134 (96.4)	13 (92.9)	6 (3.9)	0.180
	Retrospective classification for inside/outside criteria not possible		32 (17.2)	29 (15.6)	3 (17.6)	0 (0)	0.968
Operative detail	Arterial anastomosis	Primary aortic anastomosis	6 (3.2)	6 (3.6)	0 (0)	0 (0)	0.386
		Standard anastomosis	152 (82.2)	136 (81.0)	16 (94.1)		
		Complex vascular reconstruction	27 (14.6)	26 (15.5)	1 (5.9)		
	Portal vein anastomosis	End to end anastomosis	180 (97.3)	163 (97.0)	17 (100)	0 (0)	0.914
		Jump graft	2 (1.1)	2 (1.2)	0 (0)		
		Other	3 (1.6)	3 (1.8)	0 (0)		
	Bile duct anastomosis	End to end anastomosis	171 (92.4)	154 (91.7)	17 (100)	3 (1.6)	0.547
		Biliodigestive anastomosis	7 (3.8)	7 (4.2)	0 (0)		
		End to side anastomosis	4 (2.2)	4 (2.4)	0 (0)		
	Cold ischemia time (min)		594.34; 586 (313-1098)	603.51; 597.0 (336-1098)	498.06; 475.5 (313-795)	1 (0.5)	**0.003**
	Warm ischemia time (min)		50; 48 (22-181)	50.28; 48 (22-181)	47.24; 47 (35-73)	0 (0)	0.451
		PRBC (n)	7.24; 6 (0-38)	7.26; 6 (0-38)	7; 5 (0-21)	0 (0)	0.924
		FFP (n)	11.31; 10 (0-35)	11.46; 10 (0-35)	9.88; 8 (4-24)		0.483

	Intraoperative transfusion requirement	TC (n)	1.28; 1 (0-6)	1.28; 1 (0-6)	1.29; 1 (0-4)		0.944
	Intraoperative resuscitation		7 (3.8)	7 (4.2)	0 (0)	0 (0)	1.000
	Operative time (min)		230.13; 220 (114-573)	231.39; 220 (114-573)	217.71; 216 (138-336)	0 (0)	0.457
	Split liver graft		7 (3.8)	7 (4.2)	0 (0)	0 (0)	1.000
	Graft weight (g)		1770.1; 1759.5 (833-3074)	1757.19; 1750.5 (833.0-3074.0)	1903.88; 1883.5 (1350.0-2662.0)	3 (1.6)	0.188
	Liver weight (g)		1569.3; 1517.0 (553.3-3327.0)	1574.36; 1523.0 (553.3-3327.0)	1519.88; 1455.0 (1050.0-2359.0)	2 (1.1)	0.636
	Donor recipient gender mismatch		80 (43.2)	72 (42.9)	8 (47.1)	0 (0)	0.800
Perioperative outcome	Hemodialysis during stay		45 (24.3)	44 (26.2)	1 (5.9)	0 (0)	0.077
	Hemodialysis at discharge		3 (1.6)	3 (1.8)	0 (0)	1 (0.5)	1.000
	Wound infection within 30 days		5 (2.7)	5 (3.0)	0 (0)	0 (0)	1.000
	Rejection within 90 days	Overall	23 (12.4)	20 (11.9)	3 (17.6)	0 (0)	0.449
		Antibody mediated	0 (0)	0 (0)	0 (0)		
		Cellular	23 (12.4)	20 (11.9)	3 (17.6)		
	Time on IMC / ICU		18.17; 9.5 (3-161)	18.87; 10 (3-161)	12; 9 (3-33)	19 (10.3)	0.755
	Death on IMC / ICU		20 (10.8)	20 (11.9)	0 (0)	0 (0)	0.224
	Postoperative transfusion requirement	PRBC within 48 hours (n)	6.72; 4 (1-52)	6.78; 4 (1-52)	6.29; 4 (2-21)	66 (35.7)	0.997
		FFP within 48 hours (n)	8.68; 5 (1-57)	8.90; 5 (1-57)	6.62; 4 (2-18)	48 (25.9)	0.293
		Number TC within 48 hours	2.9; 2 (1-10)	3.09; 2 (1-10)	1.63; 2 (1-2)	124 (67.0)	**0.027**
	Primary nonfunction (PNF)		19 (10.3)	18 (10.7)	1 (5.9)	34 (18.4)	0.694
	Early allograft dysfunction (EAD)		103 (55.7)	95 (56.5)	8 (47.1)	5 (2.7)	0.442
	Retransplantatio n	Within 7 days	9 (4.9)	10 (6.0)	1 (5.9)	0 (0)	0.588
		Within 90 days	15 (8.1)	14 (8.3)	1 (5.9)	4 (2.2)	1.000
		Overall	16 (8.6)	15 (8.9)	1 (5.9)	0 (0)	1.000
		Days until retransplantation	65.19; 4.5 (1-735)	69.4; 6 (1-735)	2; 2 (2-2)	0 (0)	0.381
	Reason for retransplantatio n	Vascular	3 (1.6)	3 (1.8)	0 (0)	2 (1.1)	0.911
		Initial / Primary nonfunction	10 (5.4)	9 (5.4)	1 (5.9)		
		Biliary complications	1 (0.5)	1 (0.6)	0 (0)		
		Other	1 (0.5)	1 (0.6)	0 (0)		
	Death during observational period		82 (44.4)	67 (39.9)	15 (88.2)	0 (0)	**<0.01**
Histopathological examination of explanted liver	Portal vein thrombosis		21 (11.4)	17 (10.4)	4 (23.5)	18 (9.7)	0.062
	Liver cirrhosis	Overall	176 (95.1)	163 (97.0)	13 (76.5)	7 (3.8)	0.152
		Micronodular	109 (58.9)	103 (61.3)	6 (35.3)	57 (30.8)	**0.041**
		Macronodular	42 (22.7)	36 (21.4)	6 (35.3)		0.079
	No proof of malignancy		59 (31.9)	54 (32.1)	5 (29.4)	0 (0)	1.000
	Number of tumors		1.94; 1 (1-20)	1.87; 2 (1-8)	2.91; 1 (1-20)	8 (6.1)	0.123
	Tumor size (cm)		2.95; 2.85 (0.4-9.5)	2.85; 2.6 (0.4-9.5)	3.62; 3.95 (1.2-5.3)	8 (6.1)	**0.029**
	Tumor necrosis present		76 (58.0)	64 (56.1)	9 (75.0)	6 (4.6)	0.197
	Vascular invasion	Overall	13 (10.5)	10 (8.8)	3 (17.6)	2 (1.6)	0.091
		Microvascular	11 (8.9)	9 (8.0)	2 (11.8)	3 (2.4)	0.256
		Macrovascular	1 (0.8)	0 (0)	1 (5.9)	4 (3.2)	0.090
	T status	T1	72 (57.1)	65 (57.0)	7 (58.3)	0 (0)	1.000
		T2	48 (38.1)	45 (39.5)	3 (25.0)		0.533
		T3	5 (4.0)	3 (2.6)	2 (16.7)		0.071
		T4	1 (0.8)	1 (0.9)	0 (0)		1.000
	N status	N0	66 (52.4)	60 (52.6)	6 (50.0)	0 (0)	1.000
		N1	1 (0.8)	1 (0.9)	0 (0)		1.000
		Nx	59 (46.8)	53 (46.5)	6 (50.0)		1.000
	M status	M0	126 (100)	114 (100)	12 (100)	0 (0)	n.a.
	G Status	G1	8 (6.3)	7 (6.1)	1 (8.3)	21 (16.7)	0.562
		G2	88 (69.8)	81 (71.1)	7 (58.3)		0.509
		G3	9 (7.1)	6 (5.3)	3 (25.0)		**0.040**

	Resection margins	R0	126 (100)	114 (100)	12 (100)	0 (0)	n.a.
	L status	L0	61 (48.4)	57 (50.0)	4 (33.3)	65 (51.6)	0.367
	V status	V0	116 (92.1)	107 (93.9)	9 (75.0)	0 (0)	0.054
		V1	9 (7.1)	7 (6.1)	2 (16.7)		0.205
		V2	1 (0.8)	0 (0)	1 (8.3)		0.095
	Pn status	Pn0	41 (32.5)	38 (33.3)	3 (25.0)	85 (67.5)	0.750
